# Juvenile sanctions for young adult offenders in the Netherlands: an opportunity for rehabilitation?

**DOI:** 10.1186/s13034-025-00888-3

**Published:** 2025-03-28

**Authors:** Lise J. C. Prop, André M. van der Laan, Marinus G. C. J. Beerthuizen, Charlotte S. Barendregt, Chijs van Nieuwenhuizen

**Affiliations:** 1 Research and Data Centre (WODC), Ministry of Justice and Security, PO BOX, 20301, 2500 EH The Hague, The Netherlands; 2 TRANZO, Scientific Center for Care and Welfare, Tilburg University, PO BOX 90153, 5000 LE Tilburg, The Netherlands; 3 Department of Child Law and Health Law, Leiden Law School, PO BOX 9520, 2300 RA Leiden, The Netherlands; 4 Health and Youth Care Inspectorate, Ministry of Health, Welfare and Sport, PO BOX 2518, 6401 DA Heerlen, The Netherlands; 5 GGzE Centre for Child and Adolescent Psychiatry, PO BOX 909, (DP 8001), 5600 AX Eindhoven, The Netherlands

**Keywords:** Adolescent criminal law, Juvenile sanctions for young adults, Rehabilitation, Quasi-experimental design, Propensity score matching

## Abstract

**Background:**

To improve rehabilitation and reduce recidivism, adolescent criminal law provides tailor-made sanctioning in which the emphasis is on the offender’s development. This results in the possibility that juvenile sanctions, in which education, treatment and rehabilitation are central, can be applied to young adult offenders. It is unknown, however, whether there is a relationship between the developmental focus of adolescent criminal law and the rehabilitation of young adult offenders. Therefore, the aim of this study was to examine whether juvenile sanctions are efficacious in rehabilitating young adult offenders.

**Methods:**

A quasi-experimental design was used with judicial observational data. From a total of young adults (*n* = 671) sentenced with juvenile sanctions and young adults (*n* = 7.221) sentenced with adult sanctions different subsamples were composed. The subsamples were based on distinct starting conditions: (1) young adults engaged in education or employment, (2) young adults without education, income or on unemployment benefits, (3) young adults living independently, and (4) young adults living with parents or institutionalized. Rehabilitation was operationalized as changes or stability in education/employment status and housing conditions two years after the sanction was imposed. Binary logistic regression analyses were used to assess the associations. *Results:* Young adults who were engaged in education or employment before their conviction were more likely to maintain this status after a juvenile sanction (*OR* = 1.43, *p* < 0.05) than after an adult sanction. Young adults who were not engaged in education or employment had a lower chance (*OR* = 0.677, *p* < 0.05) of improving their status after a juvenile sanction than after an adult sanction. No significant differences were found for housing conditions.

**Conclusions:**

By focusing on maintaining existing engagement in education and employment, juvenile sanctions align with the developmental needs of young adults and promote stability in their transition to mature societal roles. This study highlights the importance of reinforcing existing prosocial bonds and providing adequate support for those needing to establish new prosocial bonds.

## Introduction

Young adulthood is a period characterized by important transitions to more mature roles, which are both theoretically and empirically associated with a decrease in recidivism and desistance from crime [1, 15, 19, 22, 25, 26]. These more mature roles involve, for example, the transition to higher education, being employed, living independently and having suitable housing conditions. However, young adults often face significant challenges in successfully transitioning into these roles. For those involved in criminal behavior, these challenges are exacerbated by backgrounds of social and economic disadvantage, such as limited educational opportunities, unstable family environments, and inadequate support systems. Such disadvantages hinder their ability to establish the stability necessary for desistance from crime. Given these difficulties, it is particularly important for young adults to retain and strengthen mature roles and conventional social bonds, as these connections are crucial for their reintegration. However, sanctions can disrupt these bonds, making desistance even more difficult. This highlights the importance of tailored interventions for young adults that focus on facilitating the transition into these more mature roles and maintaining them [5, 8, 16, 19, 37].

Since 2014, adolescent criminal law in the Netherlands has provided a tailor-made approach to the sentencing of young adult offenders. The emphasis is on the offender’s development. Juvenile sanctions, in which education, treatment and rehabilitation are central, can now also be applied to young adult offenders aged 18 up to and including 22 [7, 36]. The main assumption of adolescent criminal law is that juvenile sanctions are more suited for young adults, as a positive behavioral change can be accomplished given the offender’s development (Parliamentary Documents II, 2012/13, 33,498, no. 3 [30]. It remains unknown, however, whether there is a relationship between the developmental focus of adolescent criminal law and its effects on the rehabilitation of young adult offenders.

To understand why some individuals continue their delinquent behavior while others desist from crime during (young) adulthood, Sampson and Laub [35] developed an age-graded theory of informal social control. According to this theory, the importance of informal social bonds to society (i.e., work, family, school and community) at all ages is emphasized and associated with rehabilitation and a decrease in recidivism. According to Sampson and Laub [35], individuals are more likely to commit crimes when bonds to society are weak or broken. Establishing and retaining prosocial bonds can lead to desistance from crime. Furthermore, human agency is considered important for desistance from crime [10, 19, 28]. The idea of human agency in the context of desistance is that individuals make a conscious choice to stop committing crimes and act accordingly. Farrall and colleagues [10] described this process as a development from social exclusion to social inclusion through establishing prosocial bonds. Social exclusion can occur when an individual does not participate in society’s main activities, such as education or employment.

Previous research shows that characteristics of rehabilitation, such as engagement in education or employment, living independently and having suitable housing conditions, are associated with desistance from crime [5, 8, 12, 37]. Varying results have been found regarding the relationship between education and recidivism [2, 5, 20, 29]. A study on the role of school and the rehabilitation of adolescent offenders [20] revealed that adolescents who went to school shortly after prison release were less likely to reoffend. In a meta-analysis by Assink and colleagues [2], a relationship between negative school characteristics (e.g., dropping out of school) and recidivism was found. However, Loeber and colleagues [21] did not find an association between school dropout and persistence or desistance from crime.

Work has both immediate and gradual negative effects on delinquency [17, 19, 37]. In addition, it has been found that being employed has a greater negative effect on committing crimes compared to being on (unemployment) benefits [39]. Work provides immediate social control, and individuals have less time to commit crimes [17]. Being employed enables an individual to establish prosocial bonds and the ability to obtain responsibility. Therefore, the acquisition of work and job stability are both important for the rehabilitation process [19, 37].

Furthermore, studies on the role of parental support in young adulthood show that both practical, i.e., providing suitable housing and financial support, and emotional support can be associated with a decrease in recidivism and desistance from crime [8, 9, 14, 18]. Although practical support can be associated with desistance from crime during young adulthood, prior research also shows that being financially dependent on one’s parents may undermine young adults’ sense of autonomy [16, 42]. In addition, young adults who spent more time in adult roles, i.e., being employed and/or living independently from their parents, reported less engagement in delinquency [15].

### The current study

Rehabilitation is considered important for young adult offenders. Previous studies, however, have focused mainly on recidivism as a central outcome measure of judicial interventions [34]. The current study aims to address this gap by focusing on rehabilitation as an outcome measure of judicial interventions, emphasizing the development and retention of prosocial bonds─a key construct in understanding desistance from crime [35]. Prosocial bonds refer to the connections that individuals establish and maintain with societal institutions, which foster inclusion and social integration [10, 19]. Education, employment and stable housing are foundational bonds, providing individuals with the skills and opportunities needed to integrate successfully into society [8, 15, 20].

In the current study, a quasi-experimental research design with longitudinal (judicial) observational data at the individual level was used. To bridge the theoretical framework with empirical analysis, this study uses stability and change in education, employment and housing conditions as key outcomes of juvenile sanctions for young adult offenders. These outcomes are not only theoretically grounded in the age-graded theory of informal social control [35] but are also supported by empirical findings linking them to a decrease in recidivism and desistance from crime [5, 17, 37].

The research questions are as follows:

1. To what extent do juvenile sanctions for young adult offenders lead to a change or stability in education or employment status two years after the sanction?
2. To what extent do juvenile sanctions for young adult offenders lead to a change or stability in housing conditions two years after the sanction?

## Methods

### Data

Data from five sources were linked on an individual level. First, data regarding (offending) age, type of sanction, and registration date of the criminal case were obtained from the registration system of the Dutch Public Prosecution Service (RAC-min). Second, the Judicial Information System (JIS), a database containing information regarding the criminal history of offenders, was used. Data regarding criminal careers, the characteristics of the offender and the settlement of the criminal case were obtained from this database. Third, the Social Statistics Database from Statistics Netherlands was used to obtain data regarding rehabilitation, such as education, employment and housing conditions [4]. Fourth, information regarding custodial sentences (i.e., admission and release dates) was obtained from the Dutch prison registration system. Finally, data regarding community service and probation supervision were obtained from the Integral Probation Information System of the Dutch probation service.

### Sample

Criminal cases of offenders aged 18 to 22 years at the time of committing the crime, attempted between April 2014 and December 2015, were selected from the registration system of the Dutch Public Prosecution Service. Within the cohort, n = 842 criminal cases were settled in juvenile court, while n = 21,284 criminal cases were settled in adult court. For this study, we used the same initial sample as [31]. Various selections were made for this initial sample, which reduced the sample size for subsequent analysis (see Table 1). Since RAC-min only provides information on the criminal case level, all cases were linked to the Judicial Information System (JIS). This second step enabled access to data on individual level, including personal identification numbers, which allowed for linkage with the Social Statistics Database from Statistics Netherlands. After this step, the number of criminal cases within the group of juvenile sanctions increased to n = 904. The Judicial Information System identified some cases that were initially identified as settled in adult court, as criminal cases settled in juvenile court. All cases that were identified as settled in juvenile court by RAC-min and/or JIS were included in the group of young adults sentenced with juvenile sanctions.

**Table 1:** Selection procedure for cases with juvenile sanctions and adult sanctions

	Juvenile sanction	Adult sanction	Total
RAC-min^a^ selection	842	21,284	22,126
Linking JIS^b^	904	19,216	20,120
Remove duplicate cases and cases with discrepancies	866	16,133	16,999
Remove cases from AS-group^c^ with case in JS-group^d^	866	15,843	16,709
Remove unknown district	865	14,579	15,444
Remove less relevant cases	858	14,001	14,859
Remove less relevant sanctions	787	10,644	11,431
Remove cases completed after 2017	720	8,737	9,457
Select first criminal case of individual	671	7,221	7,892

^a^The registration system of the Dutch Public Prosecution Service

^b^The Judicial Information System

^c^Young adults sentenced with juvenile sanctions

^d^Young adults sentenced with adult sanctions

To prevent self-matching, all adult court criminal cases of individuals with juvenile court criminal cases were removed from the subset. Hence, juvenile court cases of individuals could no longer be matched with their own adult court cases. Furthermore, cases involving less relevant offenses for juvenile criminal law, such as traffic offenses and misdemeanors, and cases involving less relevant sanctions (i.e., fines and other settlements) were removed. Due to the two-year observation period for measuring change or stability in rehabilitation, only criminal cases with a start of observation before 2017 were included in the sample. All criminal cases with a sanction continuing in or after 2017 were excluded. This encompasses cases involving adult detentions exceeding two years, a disposal to be treated on behalf of the state (in Dutch tbs-maatregel) for adults or a placement in a youth facility (in Dutch: PIJ-maatregel) for juveniles. Notably, adult detention and a tbs-order may have an indefinite duration, while placement in a youth facility can last up to seven years. These cases were removed because it was not possible to observe and measure change or stability in rehabilitation two years after the sanction given the timeline. This resulted in n = 671 young adults being sentenced with juvenile sanctions and n = 7221 young adults being sentenced with adult sanctions.

### Operationalization

In this study, we conceptualized rehabilitation as change or stability in prosocial bonds to society and the presence of adult roles after a sanction. To this end, the situation regarding education, employment and housing conditions immediately before the registration of the criminal case at the Public Prosecution Service and 2 years after a sanction was determined.

When an individual was registered as a student or had an income from work, it was classified as having prosocial bonds. When an individual was not registered as a student, had no income or was on (unemployment) benefits, it was classified as having no prosocial bond. For housing conditions, it was assumed that both young adults living with their parents and young adults living in an institution received (some) pedagogical support and spent less time in adult roles than young adults living independently [15]. Therefore, when an individual was living independently (with or without a partner or roommates), it was classified as having adult roles. When an individual lived with his parents or was institutionalized, it was classified as the absence of adult roles.

### Outcome measures

Two categories were distinguished for both education or work and housing conditions. When an individual was engaged in education or employment, the case was assigned to the first category. When an individual was not engaged in education, was unemployed or was on (unemployment) benefits, the case was assigned to the second category. Regarding housing conditions, the first category applies when an individual was living independently (with or without a partner or roommates). The second category applies when an individual was living with his parents or was institutionalized. Changes or stability in education or work and housing conditions were measured at two points: immediately before the registration of the criminal case at the Public Prosecution Service (T0) and two years after the completion of the imposed sanction (T1).

Changes or stability in education or work and housing conditions may indicate a possible positive or negative effect of juvenile sanctions for young adults on rehabilitation. Regarding education and work, a positive effect (i.e., positive stability or positive change) is reported when an individual was attending education or had a job at T1 (see Table 2). A negative effect (i.e., negative stability or negative change) was reported when an individual did not attend education or had no job at T1. Regarding housing conditions, a positive effect (i.e., positive stability or positive change) was reported when an individual was living independently (with or without a partner or roommate; see Table 3). A negative effect (i.e., negative stability or negative change) was reported when an individual was not living independently (e.g., with parents or in an institution). The observation period in this study was two years (i.e., 730 days). The start of the observation period was directly after the sanction was terminated.

**Table 2:** Possible outcomes for stability and change in education or work

	Two years after finishing sanction (T1)	
Before registration of criminal case (T0)	Education or job	No education or job
Education or job	Positive stability	Negative change
No education or job	Positive change	Negative stability

Positive stability or positive change refers to a positive effect, negative stability or negative change refers to a negative effect

**Table 3:** Possible outcomes for stability and change in housing conditions

	2 years after finishing sanction (T1)	
Before registration of criminal case (T0)	Living independently	Not living independently
Living independently	Positive stability	Negative change
Not living independently	Positive change	Negative stability

Positive stability or positive change refers to a positive effect, negative stability or negative change refers to a negative effect

### Propensity score matching

Propensity score matching is a statistical technique used to reduce selection bias in observational studies [32, 33]. The propensity score represents the estimated likelihood that an individual is assigned to the group of young adults sentenced with juvenile sanctions. By matching treated and untreated individuals with similar propensity scores, propensity score matching mimics randomization. The rationale for propensity score matching lies in addressing confounding, which can arise when treatment allocation is not random but influenced by observable factors [3]. Unlike randomized controlled trials, observational studies lack random assignment, making it difficult to separate treatment effects from effects of confounders. Propensity score matching attempts to close this gap by creating matched samples where covariate distributions are similar between treatment and control groups, thereby approximating the conditions of a randomized controlled trial [23]. We used a logistic regression model to estimate the propensity score. Nearest-neighbor matching with a caliper of 0.05 was applied.

To assess the success of the matching process, differences in covariates between the groups were calculated using the standardized bias, which is defined as Cohen’s d for continuous variables and Cohen’s h for dichotomous variables [33]. A standardized bias exceeding 20 indicates an imbalance in characteristics. The matching results are discussed in the results section.

### Covariates in the matching

A large set of covariates were included in the propensity score matching. These fourteen covariates were selected to control for both factors related to the probability of sentencing with juvenile sanctions and the outcome measures (i.e., employment or education, and housing conditions). The inclusion of these covariates ensures that comparisons between groups were based on similar baseline characteristics, minimizing selection bias from unobserved confounders [23]. The selection of covariates was guided by legislative criteria and empirical evidence. For instance, the Explanatory Memorandum specifies that juvenile sanctions are typically eligible for offenders of serious crimes and chronic young adult offenders (Parliamentary Documents II, 2012/13, 33,498, no. 3). This is in line with previous research, which has shown that young adults who are sentenced with juvenile sanctions committed more often offences of a serious nature, were on average younger (age at the time of committing the crime and at the time of their first crime) and commit more crimes in their criminal career than young adults who are sentenced with adult sanctions [30]. Furthermore, according to previous research, prosocial bonds such as attending education, work and/or suitable housing conditions are characteristics of rehabilitation that are associated with a decrease in recidivism and desistance from crime [5, 8, 16, 19].

In this study, covariates were grouped into three categories: (1) socio-demographic covariates, (2) criminal case covariates, and (3) criminal career covariates. Socio-demographic covariates included gender, migration background, highest level of education pursued, attending education or being employed, and housing conditions. Criminal case covariates included age at the time of committing the crime, start of the criminal case in number of days since the introduction of adolescent criminal law, maximum possible sentence, type of crime, district, and age at the time of committing the offence. Criminal career covariates included age at the time of the first criminal case, total number of criminal cases, conviction density and the average maximum possible sentence.

Importantly, education, employment, and housing conditions were deliberately excluded as covariates in the matching process for the analyses where these characteristics were the primary outcome measure.

### Analyses

To measure the effect of juvenile sanctions on changes in or the stability of rehabilitation indicators 2 years after the sanction, separate analyses were performed for employment/education and housing conditions. The following steps were taken for both outcome measures (i.e., employment/education and housing conditions).

First, participants were selected from the full sample, based on their initial status regarding employment/education at T0 (i.e., right before the registration of the criminal case at the Public Prosecution Service). These participants were then divided into two groups (1) all participants that were engaged in education or employment and (2) all participants that were not engaged in education, unemployed or were on (unemployment) benefits. The same has been done for housing conditions, where group (1) consists of participants that were living independently (with or without a partner or roommates) and group (2) consists of participants that were living with their parent(s) or institutionalized.

Second, within each condition at T0, experimental participants (i.e., juvenile sanctions) were then matched with appropriate control participants (i.e., adult sanctions). This matching resulted into several smaller selections of matched experimental and control participants: (1) participants that were engaged in education or employment at T0, (2) participants that were not engaged in education, unemployed or were on (unemployment) benefits at T0, (3) participants that that were living independently (with or without a partner or roommates) at T0, and (4) participants that were living with their parent(s) or institutionalized at T0.

Third, binary logistic regression analyses were performed separately for each group, with sanction type (i.e., juvenile or adult sanction) as the independent variable, and the relevant outcome variable (i.e., employment/education and housing conditions) on T1 as the dependent variable. More specifically, analysis examined whether participants with juvenile sanctions were more likely to exhibit a positive change than negative stability compared to matched controls with adult sanctions, or whether participants with juvenile sanctions were more likely to exhibit positive stability than negative change compared to matched controls (see Table 2, for the possible outcomes on stability and change).

## Results

### Initial sample

Table 4 shows the initial unmatched sample characteristics, dividing between young adults sentenced with juvenile sanctions (JS) and young adults sentenced with adult sanctions (AS), with percentages (%) for categorical variables, and means (M) for continuous variables. Differences between young adults sentenced with juvenile sanctions and young adults sentenced with adult sanctions were tested using Chi-square tests for categorical variables and independent samples T-tests for continuous variables. The level of significance was set at *p* < 0.05, tested two-sided. As Table 4 shows, young adults sentenced with JS differed statistically significant from young adults sentenced with AS on various characteristics. First, with regard to socio-demographics the percentage of males with JS was significant higher compared to young adults with AS (χ2 (1) = 7.2, *p* < 0.05). Furthermore, both groups differed significantly in migration background (χ2 (3) = 17.7, *p* < 0.05), level of education (χ2 (6) = 233.2, *p* < 0.05), and housing conditions (χ2 (4) = 96.3, *p* < 0.05). Significant differences were also found regarding criminal case characteristics, where young adults sentenced with JS committed more often violent property crimes (χ2 (1) = 196.0, *p* < 0.05) and sexual offenses (χ2 (1) = 22.0, *p* < 0.05), while drug-related crimes were less common in this group (χ2 (1) = 7.7, *p* < 0.05). Both groups differed also in type of imposed sanction (χ2 (2) = 92.6, *p* < 0.05), court district (χ2 (10) = 47.4, *p* < 0.05), and number of days since the introduction of adolescent criminal law (*t*(799,6) = -8,09, *p* < 0.05). Finally, significant differences were found regarding criminal career characteristics. Both groups differ significantly on the average age at the start of the criminal career (*t*(856) = 7.7, *p* < 0.05), average maximum penalty in years (*t*(856) = 7.7, *p* < 0.05), conviction density (*t*(783) = -5.4, *p* < 0.05). These differences underscore the importance of matching to create comparable groups.

**Table 4:** Characteristics of young adults sentenced with juvenile or adult sanctions before matching

	Juvenile sanction	Adult sanction
	(*n* = 671)	(*n* = 7221)
	%/*M*	%/*M*
Sociodemographic characteristics		
Male (%)*	92.3	88.9
Migration background (%)*		
Netherlands	50.2	42.6
Morocco/Turkey	21.5	21.9
Surinam/Neth. Antilles and Aruba	10.3	13.8
Other	18.0	21.7
Highest level of education pursued (%)*		
Primary or lower secondary	45.3	21.5
Community college 1	4.0	4.1
Community college 2	29.1	28.6
Community college 3	6.1	10.4
Community college 4	8.9	17.8
Higher secondary or university	1.9	5.5
Unknown	4.6	12.2
Working/schooling participation (%)*		
Employed	10.3	19.9
Unemployment benefits	32.0	23.8
Student	38.2	26.7
No registered income	19.5	29.5
Type of household (%)*		
With parents	60.4	55.2
Independent	17.6	19.6
Independent with housemate(s)	6.3	9.8
Institutionalized	11.3	4.4
Unknown	4.5	11.0
Criminal case characteristics (M)		
Age at time of committing the crime*	19.4	20.7
Start of criminal case since introduction ACL. in days*	336.6	277.0
Maximum possible sentence	5.8	4.6
Type of crime (%)		
Violent crime	31.0	29.5
Drug crime	6.0	9.1
Sexual crime	3.0	1.0
Violent property crime	17.4	4.5
Non-violent property crime	40.8	41.3
Vandalism and public disturbance	20.6	23.5
Other	9.2	10.6
Court District (%)		
Amsterdam	6.1	7.8
North Holland	5.4	8.6
Central Netherlands	10.7	11.1
North Netherlands	13.4	9.2
The Hague	8.0	14.0
Rotterdam	18.6	14.7
Limburg	6.9	5.9
East Brabant	6.1	5.7
Zealand West Brabant	6.3	6.7
Gelderland	12.2	10.4
Overijssel	6.3	5.7
Criminal career characteristics (M)		
Age at time first criminal case	16.3	17.1
Total number of criminal cases	3.6	3.4
Conviction density	1.2	1.0
Average maximum possible sentence	5.1	4.4

^*^p <.05

### Matching results

As shown in Tables 5, 6, 7, 8, it was not possible to match all young adults sentenced with juvenile sanctions in the selected subsamples. Among the group of young adults sentenced with juvenile sanctions who were engaged in education or employment, *n* = 307 (94.5%) were matched to young adults sentenced with adult sanctions. Among the young adults sentenced with juvenile sanctions who were not engaged in education or employment, *n* = 328 (94.8%) were matched to young adults sentenced with adult sanctions. We were able to match *n* = 153 (95.6%) of the young adults sentenced with juvenile sanctions who were living independently (with or without a partner or roommates) to young adults sentenced with adult sanctions. For 93.7% (*n* = 479) of the young adults sentenced with juvenile sanctions that were living with parents or being institutionalized, a match was found in the group of young adults sentenced with adult sanctions. A comparison between the matched and non-matched individuals showed that young adults who were not matched were involved in serious offenses (i.e., violent property crimes) and harsher sanctions (i.e., unconditional juvenile detentions), compared to the matched young adults.

**Table 5:** Pre- and post-matching characteristics of young adults attending education or having a job at T0

	Pre-matching			Post-matching		
	Juvenile sanction	Adult sanction		Juvenile sanction	Adult sanction	
	(*n* = 325)	(*n* = 3367)		(*n* = 307)	(*n* = 307)	
	%/*M*	%/*M*	%bias	%/*M*	%/*M*	%bias
Sociodemographic characteristics (%)						
Male	92.6	89.8	9.9	92.5	93.8	5.2
Migration background (%)						
Netherlands	49.8	48.2	3.2	49.5	45.3	8.4
Morocco/Turkey	20.3	20.2	0.25	21.2	21.5	0.7
Surinam/Neth. Antilles and Aruba	12.3	12.7	1.2	12.1	12.7	1.8
Other	17.5	18.9	3.6	17.3	20.5	8.2
Highest level of education pursued (%)						
Primary or lower secondary	36.0*	12.8	55.5	34.9	33.9	2.1
Community college 1	4.0	3.4	3.2	3.6	4.2	3.1
Community college 2	34.5	32.3	4.7	35.5	33.6	4.0
Community college 3	8.0*	13.9	19.1	8.1	6.8	5.0
Community college 4	13.2*	24.7	29.7	13.7	16.6	8.1
Higher secondary or university	–	–	–	–	–	–
Unknown	–	–	–	–	–	–
Type of household (%)						
With parents	69.2	68.0	2.6	70.0	66.4	7.7
Independent	12.0*	16.5	12.9	12.1	14.0	5.6
Independent with housemate(s)	5.8*	10.0	15.7	5.5	8.8	12.9
Institutionalized	9.8*	2.5	31.9	9.4	8.1	4.6
Unknown	3.1	3.1	0	2.9	2.6	1.8
Criminal case characteristics (M)						
Age at time of committing the crime	19.1	20.4	102.0	19.1	19.4	24.2*
Start of criminal case since introduction ACL. in days	326.9	278.3	26.7	321.8	321.5	0.2
Maximum possible sentence	5.9*	4.6	43.6	5.7	5.7	0
Type of crime (%)						
Violent crime	30.8	32.8	4.3	31.9	29.6	5.0
Drug crime	6.2	7.6	5.5	6.5	7.8	5.1
Sexual crime	3.7*	1.3	15.9	-	-	-
Violent property crime	17.8*	4.6	43.9	14.7	15.0	0.8
Non-violent property crime	39.1	35.1	8.3	40.1	39.7	0.8
Vandalism and public disturbance	16.6*	26.0	23.1	16.6	19.2	6.8
Other	8.3	10.2	6.6	8.8	8.8	0
Court District (%)						
Amsterdam	10.2	8.0	7.7	10.4	10.4	0
North Holland	3.7	7.6	17.1	3.9	6.2	10.6
Central Netherlands	12.3	12.2	0.3	13.0	11.4	4.9
North Netherlands	10.5	9.5	3.3	10.7	9.1	5.4
The Hague	10.2	13.3	9.6	9.4	11.4	6.6
Rotterdam	17.2	14.9	6.3	16.6	19.9	8.6
Limburg	6.8	5.3	6.3	7.2	4.6	11.16
East Brabant	5.5	5.3	0.9	5.2	5.5	1.36
Zealand West Brabant	5.2	5.2	0	5.2	3.9	6.3
Gelderland	11.4	11.3	0.3	11.1	10.1	3.2
Overijssel	7.1	5.9	4.9	7.2	7.5	1.1
Criminal career characteristics (M)						
Age at time first criminal case	16.5*	17.4	36.5	16.5	16.6	4.3
Total number of criminal cases	3.0*	2.5	16.7	3.0	3.0	0
Conviction density	1.2*	1.0	40.0	1.2	1.1	3.2
Average maximum possible sentence	5.4*	4.4	43.9	5.2	5.3	4.1

^*^p <.05

**Table 6:** Pre- and post-matching characteristics of young adults who were not attending education had no income or were on (unemployment) benefits at T0

	Pre-matching			Post-matching		
	Juvenile sanction	Adult sanction		Juvenile sanction	Adult sanction	
	(*n* = 346)	(*n* = 3,854)		(*n* = 328)	(*n* = 328)	
	%/*M*	%/*M*	%bias	%/*M*	%/*M*	%bias
Sociodemographic characteristics (%)						
Male	91.9*	88.1	12.7	92.1	94.8	11.0
Migration background (%)						
Netherlands	50.6*	37.7	26.1	50.0*	39.0	22.2*
Morocco/Turkey	22.5	23.4	2.1	23.2	23.8	1.4
Surinam/Neth. Antilles and Aruba	8.4*	14.8	20.2	8.5	9.8	4.5
Other	18.5*	24.1	13.7	18.3*	27.4	21.8*
Highest level of education pursued (%)						
Primary or lower secondary	54.0*	29.1	51.1	53.7	46.3	14.8
Community college 1	4.0	4.7	3.4	4.3	3.4	4.7
Community college 2	24.0	25.4	3.2	24.1	21.0	7.4
Community college 3	4.3*	7.3	12.9	4.6	3.0	8.4
Community college 4	4.9*	11.7	25.2	4.9	7.9	12.3
Higher secondary or university	–	–	–	–	–	–
Unknown	–	–	–	–	–	–
Type of household (%)						
With parents	52.0*	44.0	16.0	51.8	50.9	1.8
Independent	22.8	22.3	1.2	22.9	19.5	8.3
Independent with housemate(s)	6.6	9.7	11.4	6.7	5.8	3.7
Institutionalized	12.7*	6.1	23.0	12.5*	7.3	17.6
Unknown	5.8*	17.9	38.7	6.1*	16.5	33.7*
Criminal case characteristics (M)						
Age at time of committing the crime	19.8	20.9	84.4	19.8	19.9	7.7
Start of criminal case since introduction ACL in days	345.6	275.8	38.2	343.1	336.0	3.8
Maximum possible sentence	5.7*	4.6	37.7	5.5	5.5	0
Type of crime (%)						
Violent crime	31.2	26.6	10.2	30.8	32.0	2.6
Drug crime	5.8*	10.5	17.4	5.8	6.7	3.7
Sexual crime	–	–	–	–	–	–
Violent property crime	17.1*	4.4	43.0	14.3	14.6	0.9
Non-violent property crime	42.5	46.6	8.3	43.3	40.2	6.3
Vandalism and public disturbance	24.3	21.4	6.9	24.1	24.4	0.7
Other	10.1	10.9	2.6	9.8	8.8	3.4
District (%)						
Amsterdam	2.3*	7.7	25.8	2.4	5.8	17.5
North Holland	6.9	9.5	9.5	7.3	6.1	4.8
Central Netherlands	9.2	10.1	3.0	9.5	11.0	4.9
North Netherlands	16.2*	8.9	22.3	7.3	6.1	4.8
The Hague	6.1*	14.7	28.8	6.4	9.5	11.5
Rotterdam	19.9*	14.6	14.1	19.5	17.4	5.4
Limburg	6.9	6.5	1.6	7.0	4.0	13.3
East Brabant	6.6	6.1	2.1	7.0	7.9	3.4
Zealand West Brabant	7.2	6.8	1.6	6.7	8.5	6.8
Gelderland	13.0*	9.6	10.8	12.8	12.2	1.8
Overijssel	5.5	5.5	0	5.5	4.6	4.1
Criminal career characteristics (M)						
Age at time first criminal case	16.1*	16.8	26.3	16.1	16.3	8
Total number of criminal cases	4.2	4.2	0	4.2	4.3	2.4
Conviction density	1.2	1.1	16.7	1.2	1.2	0
Average maximum possible sentence	4.9*	4.4	25.0	4.8	4.7	4.6

^*^p <.05

**Table 7:** Pre- and post-matching characteristics of young adults who were living independently at T0

	Pre-matching			Post-matching		
	Juvenile sanction	Adult sanction		Juvenile sanction	Adult sanction	
	(*n* = 160)	(*n* = 2,124)		(*n* = 153)	(*n* = 153)	
	%/*M*	%/*M*	%Bias	%/*M*	%/*M*	%Bias
Sociodemographic characteristics (%)						
Male	86.9	82.1	13.3	86.3	86.8	1.5
Migration background						
Netherlands	55.0	46.3	17.4	56.2	53.6	5.2
Morocco/Turkey	13.1	16.1	8.5	13.7	15.7	5.7
Surinam/Neth. Antilles and Aruba	12.5	17.1	13.0	11.8	11.8	0
Other	19.4	20.4	2.5	18.3	19.0	1.8
Highest level of education pursued						
Primary or lower secondary	56.3*	25.9	62.9	54.9	47.7	14.4
Community college 1	–	–	–	–	–	–
Community college 2	26.3	29.4	6.9	27.5	26.8	1.6
Community college 3	–	–	–	5.9	7.2	5.3
Community college 4	6.9*	16.2	29.7	7.2	12.4	17.6
Higher secondary or university	–	–	–	–	–	–
Unknown	–	–	–	–	–	–
Working/schooling participation						
Employed	7.5*	15.7	26.0	7.8	13.1	17.5
Unemployment benefits	50.0*	37.5	25.3	50.3	43.8	13.0
Student	28.7	26.2	5.6	28.1	28.1	0
No registered income	13.8*	20.6	18.1	13.7	15.0	3.7
Criminal case characteristics (M)						
Age at time of committing the crime	19.6	21.0	103.6	19.7	19.9	16.6
Start of criminal case since introduction ACL. in days	325.6	269.4	32.2	323.4	321.6	0.9
Maximum possible sentence	5.6*	4.4	43.5	5.5	5.6	1.8
Type of crime (%)						
Violent crime	27.5	31.6	9.0	28.1	23.5	10.5
Drug crime	5.6	8.4	11.0	5.9	7.8	7.5
Sexual crime	–	–	–	–	–	–
Violent property crime	17.5*	4.5	43.6	15.0	14.4	1.7
Non-violent property crime	46.9	42.0	9.9	48.4	5.1	5.2
Vandalism and public disturbance	21.3	23.7	5.7	20.9	16.0	12.7
Other	10.6	11.3	2.2	11.1	9.2	6.3
District (%)						
Amsterdam	–	–	–	–	–	–
North Holland	6.9	6.9	0	5.9	10.5	16.9
Central Netherlands	–	–	–	5.9	9.2	12.6
North Netherlands	18.8*	11.3	21.1	19.0	18.3	1.8
The Hague	8.1	12.8	15.5	8.5	9.8	4.5
Rotterdam	11.3	15.5	12.4	11.1	11.1	0
Limburg	6.9	6.3	2.4	–	–	–
East Brabant	8.1*	4.4	15.5	8.5	0.078	2.6
Zealand West Brabant	–	–	–	–	–	–
Gelderland	16.3	12.5	10.8	15.7	14.4	3.6
Overijssel	9.4	7.2	8.0	9.2	9.2	0
Criminal career characteristics (M)						
Age at time first criminal case	16.2*	17.0	31.2	16.2	16.3	3.0
Total number of criminal cases	3.8	3.7	2.7	3.8	3.6	3.6
Conviction density	1.1	1.0	16.7	1.1	1.1	3.7
Average maximum possible sentence criminal career	4.7	4.3	21.6	4.6	4.6	0.3

^*^p <.05

**Table 8:** Pre- and post-matching characteristics of young adults who were living with their parent(s) or institutionalized at T0

	Pre-matching			Post-matching		
	Juvenile sanction	Adult sanction		Juvenile sanction	Adult sanction	
	(*n* = 511)	(*n* = 5,097)		(*n* = 479)	(*n* = 479)	
	%/*M*	%/*M*	%Bias	%/*M*	%/*M*	%Bias
Sociodemographic characteristics (%)						
Male	93.9	91.7	8.5	93.7	96.2	11.5
Migration background (%)						
Netherlands	48.7*	41.1	15.3	48.2	42.0	12.5
Morocco/Turkey	24.1	24.3	0.5	24.4	23.6	1.9
Surinam/Neth. Antilles and Aruba	9.6	12.4	9.0	9.6	9.4	0.7
Other	17.6*	22.2	11.4	17.7*	25.1	18.1
Highest level of education pursued (%)						
Primary or lower secondary	41.9*	19.6	49.1	39.9	37.8	4.3
Community college 1	5.1*	3.8	6.3	5.2	4.6	2.8
Community college 2	29.9	28.3	3.5	30.7	27.3	7.5
Community college 3	6.3*	10.6	15.6	6.5	5.0	6.5
Community college 4	9.6*	18.4	25.7	10.0	11.5	4.8
Higher secondary or university	2.3*	4.7	13.3	2.5	3.3	4.8
Unknown	4.9*	14.6	33.8	5.2	10.4	19.7
Working/schooling participation (%)						
Employed	11.2*	21.7	28.7	11.9	11.5	1.2
Unemployment benefits	26.4*	18.2	19.8	25.7	22.5	7.5
Student	41.1*	26.9	30.1	39.7	40.3	1.2
No registered income	21.3*	33.2	26.9	22.8	25.7	6.8
Criminal case characteristics (M)						
Age at time of committing the crime	19.4*	20.5	84.4	19.4*	19.6	12.5
Start of criminal case since introduction ACL. in days	340.0	280.3	32.3	338.7	328.5	5.4
Maximum possible sentence	5.9*	4.7	39.6	5.7	5.7	0
Type of crime (%)						
Violent crime	32.1	28.6	7.6	32.2	32.6	0.9
Drug crime	6.1*	9.4	12.4	6.3	6.5	0.8
Sexual crime	3.5*	1.1	16.6	3.1	2.9	1.2
Violent property crime	17.4*	4.5	43.3	15.2	15.9	1.9
Non-violent property crime	38.9	40.9	4.1	40.7	37.0	7.6
Vandalism and public disturbance	20.4	23.5	7.5	20.7	24.0	7.96
Other	8.8	10.3	5.1	9.0	8.6	1.4
District (%)						
Amsterdam	6.8	8.6	6.8	7.1	8.4	4.9
North Holland	4.9*	9.3	17.3	5.2	6.1	3.99
Central Netherlands	12.3	11.8	1.5	12.3	11.7	1.8
North Netherlands	11.7*	8.3	11.4	11.7	10.0	5.5
The Hague	8.0*	14.6	21.1	8.4	10.2	6.2
Rotterdam	20.9*	14.4	17.1	20.7	19.0	4.3
Limburg	6.8	5.8	4.1	6.5	4.8	7.4
East Brabant	5.5	6.3	3.4	5.2	6.1	3.9
Zealand West Brabant	6.7	6.4	1.2	6.9	7.5	2.3
Gelderland	11.0	9.5	4.9	10.6	11.5	2.9
Overijssel	5.3	5.1	0.9	5.4	4.8	2.7
Criminal career characteristics (M)						
Age at time first criminal case	16.4*	17.1	26.8	16.4	16.4	0
Total number of criminal cases	3.5	3.3	5.6	3.5	3.7	5.3
Conviction density	1.2*	1.0	36.2	1.2	1.2	0
Average maximum possible sentence criminal career	5.2*	4.5	30.9	5.1	5.1	1.7

^*^p <.05

There were significant differences before matching between young adults sentenced with juvenile sanctions and young adults sentenced with adult sanctions in all four subsamples (Table 5, 6, 7, 8). Matching the subsamples created balance for almost all covariates. After matching, one significant difference remained in subsample 1, i.e., young adults attending school or having a job. Young adults sentenced with juvenile sanctions were on average three months younger than the matched group of young adults sentenced with adult sanctions (Table 5). In subsample 2—i.e., young adults without education, income or with (unemployment) benefits—significant differences remained for migration background and housing conditions. Young adults sentenced with juvenile sanctions more often had a Dutch background, and housing conditions were less often unknown compared to young adults sentenced with adult sanctions (Table 6). No significant differences remained between the matched subsamples of young adults living independently (with or without a partner or roommates) (Table 7) and young adults living with parents or institutionalized (Table 8).

### Stability and change in education or employment

Educational and employment outcomes two years after the termination of the sanction show significant differences for young adults sentenced with juvenile sanctions and young adults sentenced with adult sanctions. Young adults who were engaged in education or employed– immediately before the registration of the criminal case at the Public Prosecution Service– and who were sentenced with juvenile sanctions have a statistically significant higher chance on (remaining) education or employment two years after the completion of the imposed sanction compared to young adults sentenced with adult sanctions (*OR* = 1.43, *p* < 0.05). Within the group of young adults sentenced with juvenile sanctions, 59.6% of the young adults are (still) engaged in education or employed compared to 50.8% of the young adults sentenced with adult sanctions. Young adults who were not engaged in education or employment—immediately before the registration of the criminal case at the Public Prosecution Service—had a statistically significant lower chance of engaging in education or employment after a juvenile sanction than young adults sentenced with an adult sanction (*OR* = 0.677, *p* < 0.05). Only 17.7% of the young adults sentenced with juvenile sanctions were engaged in education or employed, and 24.1% of the young adults sentenced with adult sanctions 2 years after the completion of the sanction.

### Stability and change in housing conditions

No statistically significant differences between young adults sentenced with juvenile sanctions and young adults sentenced with adult sanctions are found regarding housing conditions. Two years after the completion of the sanction, 67.3% of the young adults sentenced with juvenile sanctions were (still) living independently, compared to 66.0% of the young adults sentenced with adult sanctions (*OR* = 1.061, *p* = 0.808). Similarly, for young adults who were living with their parents or institutionalized right before the registration of the criminal case at the Public Prosecution Service, 30,7% were (still) living with their parents or institutionalized two years after the sanction, compared to 25,3% of the young adults sentenced with adult sanctions (*OR* = 1.31, *p* = 0.062).

## Discussion

Adolescent criminal law allows for a more flexible approach to the sentencing of young adult offenders, acknowledging their development and potential for rehabilitation. This flexibility is reflected in the ability to impose juvenile sanctions that focus on education, treatment and rehabilitation rather than purely punitive sanctions. The aim of this study was to test the efficacy of juvenile sanctions on the rehabilitation of young adult offenders, particularly focusing on education, employment and housing conditions two years after the sanction. The findings indicate that juvenile sanctions have different impacts on young adults' rehabilitation, possibly influenced by already existing prosocial bonds such as education or employment.

Our findings suggest that young adults who were already engaged in education or employment prior to a juvenile sanction are more likely to maintain these positive engagements two years after their sanction than those sentenced with adult sanctions. This aligns with the age-graded theory of informal social control, which emphasizes the importance of maintaining and strengthening informal social bonds as a pathway to reduce recidivism [35]. Juvenile sanctions, with their pedagogical focus, may help preserve these prosocial bonds. Given their more retributive character, adult sanctions may have a greater risk of damaging already existing social bonds and ties to the society of young adults, increasing the risk of further criminal behavior. These findings highlight the potential benefits of juvenile sanctions for young adults in preserving prosocial bonds, which are crucial for successful rehabilitation and desistance from crime [10, 19, 28]. Educational and developmentally oriented juvenile sanctions likely contribute to preserving these prosocial bonds, as they provide a framework that supports participation in constructive activities. The importance of interventions focusing on individuals’ strengths has been emphasized in several studies, where strengths can be understood as personal or environmental characteristics that are associated with positive outcomes [6, 11, 41]. Furthermore, youth who are engaged in education or employment are more likely to successfully target their criminogenic needs during probation than youth without strengths in these areas [11].

However, for young adults who were not previously engaged in education or employment, juvenile sanctions do not significantly improve their situation. Young adults sentenced with juvenile sanctions have a statistically lower chance of gaining such engagements two years after the sanction than those sentenced with adult sanctions, though the odds ratio indicates a moderate effect. The age-graded theory of informal social control suggest that the establishment of new prosocial bonds can serve as a turning point for offenders [35]. Previous research showed that young adults sentenced with juvenile sanctions often face more problems across different domains, such as cognitive deficits, lack of social skills, and impulse control problems, compared to young adults sentenced with adult sanctions (intentionally left blank). A possible explanation for the lack of a positive effect could be that these challenges may contribute to their lower changes of gaining educational opportunities or employment. This outcome underlines the complexity and challenges of rehabilitation for young adults who lack initial prosocial engagement. Although the acquisition of work and job stability are considered important for the rehabilitation process, finding and keeping a job can be challenging for young adults with a delinquent past [34, 35, 37, 39]. Providing adequate help in obtaining and retaining a job is therefore important for young adult offenders. For example, individual placement and support (IPS) is an effective model of vocational rehabilitation for helping people obtain and retain competitive employment [40]. The focus in IPS is, among others, on rapid job search, systematic job development and individual support. The need for targeted support to help young adult offenders find and retain educational opportunities or employment is crucial, although this is challenging for those with a criminal background.

No significant differences are found in the stability or change of housing conditions after a juvenile or adult sanction. This finding suggests that the type of sanction may not play a critical role in influencing housing conditions for young adult offenders. However, important differences exist between juvenile and adult sanctions. Juvenile sanctions often emphasize treatment and education and provide more tailored support, compared to adult sanctions, which are more focused on punitive measures. One explanation for the lack of difference in housing conditions could be the presence of structural barriers faced by all young adults with a criminal history, such as stigma from landlords, limited access to affordable housing, and high rates of unemployment. These barriers likely remain unaffected by whether a juvenile or adult sanction was imposed. Stable housing conditions are important conditions for successfully re-entering the community [34]. In an evaluation of housing programs for high-risk individuals [24], it was found that the timing of achieving stability in housing in the first period after finishing the custodial sentence is important for achieving long-term house stability and preventing recidivism. Research pertaining to the Housing First Model underscores the importance of immediate access to affordable and permanent housing conditions for young people [13]. However, access to housing has additional barriers for young adults with a judicial history, such as landlords who are wary of renting to individuals with a criminal history [34]. Furthermore, unemployment, which is common among these young adults, decreases the likelihood of securing permanent housing [13, 37, 39]. Addressing these universal barriers through tailored interventions is important to improving housing conditions and supporting the rehabilitation of young adult offenders, regardless the type of sanction they received.

## Strengths and directions for future research

One of the key strengths of this study is the focus on diverse rehabilitation outcomes beyond recidivism, i.e., education, employment, and housing conditions. By examining these rehabilitation outcomes, this study provides a broader perspective on the reintegration process of young adult offenders. Additionally, the use of propensity score matching to create comparable groups is a methodological strength, as it enhances the validity of the findings by controlling for baseline differences among participants.

However, rehabilitation involves more than education, employment and housing conditions. Moreover, the quality of these prosocial bonds, which significantly affects rehabilitation outcomes, was not available in our data. Furthermore, other unobserved characteristics that may influence rehabilitation success, such as human agency, individual well-being, personal motivation and social relationships, should be considered [10, 28, 34].

Future research should expand to evaluate the success of interventions within a broader context. This involves assessing multiple rehabilitation indicators beyond recidivism, such as overall well-being, health, social relationships, and the quality of these indicators, over an extended period [34]. Such comprehensive assessments can provide deeper insights into the long-term effects of juvenile sanctions for young adult offenders.

## Conclusion

This study highlights the important role of tailor-made sanctioning of young adult offenders aiming at transitioning to more mature roles and retaining them. By focusing on maintaining existing engagements in education and employment, juvenile sanctions align with the developmental needs of young adults and promote stability in their transition to mature societal roles. However, their efficacy in initiating new prosocial engagements among those who are initially disengaged from education or employment appears limited. Additionally, the lack of impact on housing stability underlines the need for comprehensive support strategies addressing the broader challenges faced by young adult offenders. Overall, the findings of this study suggest the importance of reinforcing existing prosocial bonds and providing adequate support for those needing to establish new prosocial bonds. Future research should continue to expand the scope of rehabilitation indicators and explore long-term outcomes to enhance our understanding of the effects of juvenile sanctions for young adult offenders.

## Data Availability

No data is available due to restrictions on access to the judicial data used in this study. All analysis codes are available on request.
